# Hypoglycemic effect of *Bromelia plumieri* (E. Morren) L.B. Sm., leaves in STZ-NA-induced diabetic rats

**DOI:** 10.3389/fphar.2013.00036

**Published:** 2013-04-09

**Authors:** Adolfo Andrade-Cetto, Anamarel E. Medina-Hernández

**Affiliations:** Laboratorio de Etnofarmacología, Departamento de Biología Celular, Facultad de Ciencias, Universidad Nacional Autónoma de MéxicoCoyoacán 04510, D.F. México

**Keywords:** Ethnobotany, Ethnopharmacology, *Bromelia plumieri*, streptozotocin-nicotinamide-induced diabetic rats, type 2 diabetes, traditional medicine

## Abstract

This study confirms the hypoglycemic effects of two extracts obtained from the *Bromelia plumieri* (BP) plant in streptozotocin-nicotinamide-induced diabetic rats (STZ-NA). BP has been traditionally used in the municipality of Tlanchinol Hidalgo, Mexico, to treat type 2 diabetes. Two different BP extracts were prepared and tested. The first extract was a water extract (WE), similar to that traditionally used to make tea, and the second extract was an ethanol:water extract (EWE). The extracts (WE at 35 and 350 mg/kg, and EWE at 30 and 300 mg/kg) were tested in STZ-NA-induced diabetic rats to determine whether hypoglycemia occurred after oral administration of the extracts. Phytochemistry: Two different extracts were prepared, *n*-hexane and butanol, to determine the presence of alkaloids, terpenes and flavonoids. The extracts that were administered to the STZ-NA-induced diabetic rats produced a significant hypoglycemic effect as compared with the control group, similar to that achieved with glibenclamide. We also determined that flavonoids were the main components of BP leaves. The results presented here support the hypothesis that extracts obtained from this plant have hypoglycemic effects, which are in agreement with the traditional uses of this plant.

## INTRODUCTION

Diabetes mellitus is defined as hyperglycemia that is associated with inadequate insulin secretion, either in the presence or absence of impaired insulin action. Type 2 diabetes (T2D) is characterized by tissue insulin resistance combined with a relative deficiency in insulin secretion. An individual may present primarily with either insulin resistance or β cell deficiencies, and these abnormalities can range from mild to severe. The long-term complications of diabetes include: retinopathy with potential loss of vision, nephropathy leading to renal failure, peripheral neuropathy with risk of foot ulcers and amputation, and autonomic neuropathy that can cause gastrointestinal, genitourinary, and cardiovascular symptoms and sexual dysfunction (Expert Committee on the Diagnosis and Classification of Diabetes Mellitus, 2003).

Type 2 diabetes is a public health problem. According to the (World Health Organization [WHO], 2013), more than 347 million patients are affected by this disease worldwide. In 2010, the WHO acknowledged that this disease is a major cause of mortality in Mexico. In the 2008 Mexican health services report, diabetes was found to be the first-highest cause of mortality in Mexico (SINAIS, 2013).

Diabetic people in Mexico commonly use medicinal plants to treat T2D, with or without the concomitant use of medically prescribed hypoglycemic agents (Andrade-Cetto and Heinrich, 2005). We found that in the municipalities of Tlanchinol and Tepehuacan de Guerrero in the Mexican state of Hidalgo, the leaves of the *Bromelia plumieri *(BP) plant are used to treat T2D.

### PLANT BACKGROUND

*Bromelia plumieri* (E. Morren) L. B. Sm. Bromeliaceae, also known as *B. karatas* L. and *Karatas plumieri* E. Morren, is often referred to in Mexico by the traditional names chichipo, chiyol (Espejo-Serna et al., 2005), piñuela, aguama, and cazuela (SIIT, 2013).

*Bromelia plumieri* is a tropical herb that is distributed between 400 and 1500 m in elevation. The leaves are elongated, thick and contain sharp teeth along the margins. Inflorescence sessile at the ground level. Flowering of the plant occurs between May and October (Espejo-Serna et al., 2005). The fruits of the plant range from 5 to 10 cm long and 1 to 2 cm in diameter and have a brown epidermis that is covered by a thin ocher colored layer (Parada and Duque, 1998).

The aim of the current study was to examine the acute hypoglycemic effects of BP leaf extracts that were made with either water or a mixture of ethanol and water in streptozotocin-nicotinamide (STZ-NA)-induced diabetic rats. We also sought to characterize the main components of the plant.

## MATERIALS AND METHODS

### ETHNOBOTANY

Direct interviews regarding plant use were conducted with a plant expert from Tlanchinol, Hidalgo, named Isabel Escalante, who reported that the plant grows in San Simon, Hidalgo. This information was confirmed by the inhabitants of the location, and samples were collected *in situ*. The identity of the samples was confirmed, by the specialist “Ramiro Cruz Duran.” Seven Kilograms of BP leaves were collected from different locations, after which point the plant material was dried under constant conditions at 40°C, ground in an IKA Mf10 mill and stored at room temperature.

### PLANT EXTRACTS

Plant extracts were prepared to both, study their hypoglycemic effects and to investigate the basic phytochemical composition of the plant. The water extract (WE), similar to that traditionally used to make tea, was made by boiling 27.3 g of the dry plant material in 500 ml of water, followed by filtration and lyophilization. The ethanol-water extract (EWE) was prepared by adding 20 g of the plant material to 800 ml of an ethanol and water mixture (50:50). The extract was then heated at 40°C for 4 h; thereafter it was filtered three times, followed by evaporation in a Büchi rotary evaporator.

Phytochemical analysis of the plant was performed by extracting 60 g using a Soxhlet extractor with *n*-hexane (HE) followed by methanol. The dry methanol extract was partitioned with butanol and water at a 1:1 ratio, and the butanolic phase was then dried (BE), followed by evaporation of all of the extracts in a rotary Büchi evaporator. All of the extracts were kept at -4°C until use.

### TLC ANALYSIS

The water, EWE, HE, and BE extracts were analyzed using standard TLC methods. Briefly, the samples were applied to a Merck 10 mm × 10 mm 60 F254 plate containing tree solvent systems. For alkaloids, a mixture containing dichloromethane, 85: MeOH, 14: NH_4_OH (25%), 1, was used and the plate was visualized using Dragendorff’s reagent. For glycated flavonoids, a mixture containing ethyl acetate, 60: formic acid, 10: acetic acid, 10: water, 20 was used. For aglycone flavonoids, a 50 ml mixture containing chloroform, 35: acetone, 10 and formic acid 5, was used. Both plates were visualized using diphenylborinic acid. For terpenes, a 20 ml mixture containing ethyl acetate, 6: HE 14, was used. The plate was visualized using vanillin (Wagner and Bladt, 2001; Andrade-Cetto and Rubalcaba-Mares, 2012).

### ANIMALS AND INDUCTION OF EXPERIMENTAL DIABETES

Eight-week-old Wistar rats weighing 200–250 g were obtained from either the Bioterium of the School of Science or the Bioterium of the Physiological Institute (UNAM) and were acclimatized with free access to food and water for at least 1 week in an air conditioned room (25°C and 55% humidity) with a 12 h light-dark cycle prior to performing the experiments. All methods used in this study were approved by the Internal Council of the Facultad de Ciencias of the Universidad Nacional Autónoma de México. Experimental diabetes was induced as described by Masiello et al. (1998). Rats that had fasted overnight were injected intraperitoneally with 230 mg/kg nicotinamide (NA; Sigma, N3376) 15 min prior to an intravenous injection of 65 mg/kg streptozotocin (STZ) in citrate buffer (Sigma, S0130).

### BLOOD COLLECTION AND BLOOD GLUCOSE DETERMINATIONS

Animals were handled according to the National Institutes of Health Guide for the Care and Use of Laboratory Animals (Committee for the Update of the Guide for the Care and Use of Laboratory Animals, 2011). Plasma glucose concentrations were measured using an Accutrend GC and were confirmed with a Reflotron instrument (Roche). Each assay was performed in duplicate. In total, 32 μl of blood was used in each assay.

### EXPERIMENTAL GROUPS

The animals were divided into seven different groups (1–7). Each group contained 11 rats. Group 1 was the non-diabetic control group. Group 2, the diabetic control group, both groups received 6 ml/kg of a normal saline solution. Group 3 was administered 5 mg/kg of glibenclamide, a standard oral hypoglycemic agent that was dissolved in physiological NaCl solution. Groups 4 and 5 received WE (35 mg/kg and 350 mg/kg, respectively), and groups 6 and 7 received EE (30 mg/kg and 350 mg/kg, respectively). The extracts were administrated in a physiological NaCl solution.

### STATISTICAL ANALYSES

The data were analyzed using one-way ANOVA followed by Bonferroni and Fisher tests. Plasma glucose levels were expressed as the means ± S.E.M.

## RESULTS

### ETHNOBOTANY

In the studied communities, the plant was referred to by its common names: piñuela or timbiriche. The plant was deposited at the IMSS Herbarium in Mexico City under voucher number IMMSM15814. We confirmed via direct interviews that the leaves of the plant are used to treat T2D. To treat the disease, approximately 30 g of dry plant material is boiled in 1000 ml of water, which is consumed over the course of the day.

### YIELD AND PHYTOCHEMICAL COMPOSITION

**FIGURE 1:**
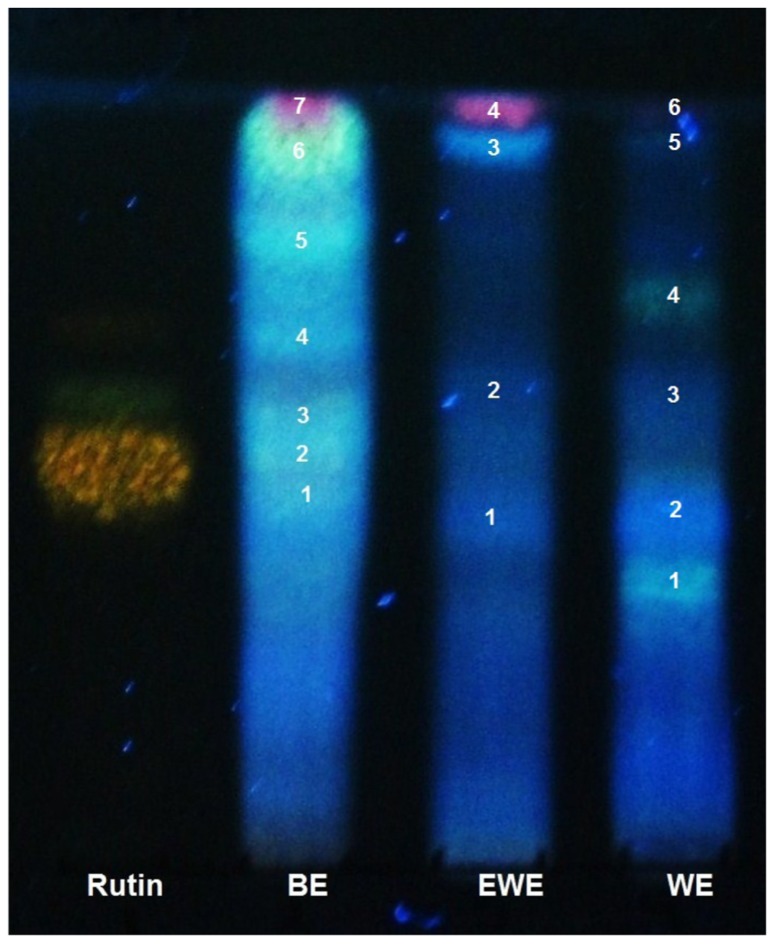
**Thin layer Chromatography of *Bromelia plumieri *leaves; BE, butanol extract, EWE, ethanol:water extract; WE, water extract**. Plates were visualized using diphenylborinic acid; the numbers indicate the detected compound.

Analysis indicated that the drug was present at a ratio of 8:1 in the WE, 9:1 in the EWE and 164:1 BE (Gaedcke and Steinhoff, 2003). Using TLC, we confirmed that the main compounds found in the plant leaves are glycosylated flavonoids, which were present in both extracts 6 (WE) and 4 (EWE), whereas seven spots were detected in the BE extract **Figure 1**. Terpenes were also detected in the WE and EWE extracts (2 spots), whereas four spots were detected in the HE. We were unable to detect the presence of alkaloids in the extracts (data not shown). In the TLC plates from the WE, EWE, and BE, we observed spots with a yellow-green coloration, which were indicative of glycosylated flavons and flavonols (Wagner and Bladt, 2001).

### ACUTE HYPOGLYCEMIC EFFECT

We confirmed that the administration of NA followed by an injection of STZ to normal rats significantly (*p* < 0.001) elevated blood glucose levels as compared with rats that were injected with citrate buffer alone. These results were similar to those reported by Masiello et al. (1998) (**Table 1**).

**Table 1:** Acute hypoglycemic effects of *Bromelia plumieri* leaves on STZ-NA-induced diabetic rats.

*Glucose (mg/dl) Groups*	*T0*	*T60*	*T120*	*T180*
*1 ND Control*	115 ± 3	120 ± 4	111 ± 4	107 ± 3
*2 D Control*	168 ± 4^3^	171 ± 5^3^	173 ± 3^3^	162 ± 4^3^
*3 D + G (5 mg/kg)*	167 ± 4	145 ± 5^2,b^	119 ± 4^3,c^	110 ± 3^3,c^
*4 D + WE (350 mg/kg)*	163 ± 3	148 ± 3^1,b,I^	142 ± 3^3,b,II^	142 ± 3^3,b^
*5 D + WE (35 mg/kg)*	173 ± 5	170 ± 9	161 ± 6^a^	148 ± 4^1,b^
*6 D + EWE (300 mg/kg)*	173 ± 4	162 ± 5	155 ± 4^2,a^	144 ± 3^2,c^
*7 D + EWE (30 mg/kg)*	167 ± 4	161 ± 3	156 ± 3^1^	147 ± 4^1,b^

*The values represent the means ± SEM. Superscript letters in the same row indicate statistical differences as compared with time 0. Superscript numbers in the same column indicate statistical differences from the control group (diabetic control group compared with non-diabetic control group). Superscript Roman numerals in the same column indicate statistical differences between the two doses of the same extract. “^a,1,I^” indicates significance at *p* < 0.05, “^b,2,II^” indicates significance at *p* < 0.01, and “^c,3^” indicates significance at *p* < 0.001. Gl, glucose; ND, non-diabetic; D, diabetic; G, glibenclamide; EWE, Ethanol:water extract; WE, water extract.*

In the diabetic rats, the positive control glibenclamide showed a hypoglycemic effect from 60 through 180 min as compared to the control group at time 0. The plant extracts also displayed significant hypoglycemic effects (**Table 1**).

Rats orally treated with a 35 mg/kg dose of the WE demonstrated hypoglycemia after 120 min which remained significant until 180 min as compared with the time 0. At the 350 mg/kg dose, the rats that had been treated with the WE had hypoglycemia from 60 to 180 min compared with the control group and time 0. The WE exerts an early hypoglycemic effect at the 350 mg/kg dose as compared with the 35 mg/kg dose. Maximal effects of the WE were observed after 180 min of treatment (**Table 1**).

Treatment of the diabetic rats with 30 mg/kg of the EWE led to significant decreases in plasma glucose levels from the 120 min time point through the 180 min time point as compared with the control group. At doses of 300 mg/kg, there was a significant effect from 120 through 180 min as compared to the with the control group and time 0. Treatment with 300 mg/kg doses also produced a robust effect as compared with the 30 mg/kg treated group. Maximal activity of the ethanol-WE was observed after 180 min.

These results support acceptance of the null hypothesis that there are no significant differences between treatment with the tested plant extracts and glibenclamide, a standard hypoglycemic drug.

## DISCUSSION

In Mexico, people who suffer from T2D often use medicinal plants to treat the disease, with or without concomitant medical intervention (Andrade-Cetto, 2010). This phenomenon reinforces the importance of the study of traditionally used hypoglycemic plants, because not all of the plants used induce the desired effect. In the present study, BP exerted a statistically significant hypoglycemic effect. In the rat model used in the present study, the injection of NA followed by STZ increased glucose levels in rats as compared with the non-diabetic control group. Glibenclamide at 5 mg/kg decreased these levels, indicating that the STZ-NA model is an adequate model that can be used to test the efficacy of hypoglycemic plants. Treatment with the WE of *Bromelia* at 350 mg/kg produced the best hypoglycemic effect. Traditionally, the plant preparation is boiled and the infusion is consumed over the next several hours. In the present study, we found that this dose (35 mg/kg) reduces glucose levels after 120 min. However, a dose of 350 mg/kg produced a superior effect. We also found that the main components of the extracts are flavons and flavonols. These compounds are known to produce hypoglycemic effects, as previously demonstrated by Torres-Piedra et al. (2010).

Altogether, the results of the present study demonstrate the hypoglycemic effects of BP**and support**the traditional use of the plant for the treatment of T2D.

## Conflict of Interest Statement

The authors declare that the research was conducted in the absence of any commercial or financial relationships that could be construed as a potential conflict of interest.
